# A Comparative Study of Participation in Physical Education Classes among 170,347 Adolescents from 54 Low-, Middle-, and High-Income Countries

**DOI:** 10.3390/ijerph17155579

**Published:** 2020-08-02

**Authors:** João Martins, Adilson Marques, Miguel Peralta, Duarte Henriques-Neto, João Costa, Marcos Onofre, Miguel González Valeiro

**Affiliations:** 1 Facultad de Ciencias del Deporte y la Educación Física, Universidad de A Coruña, 15001 A Coruña, Spain; maglez@udc.es; 2 Laboratório de Pedagogia, Faculdade de Motricidade Humana e UIDEF, Instituto de Educação, Universidade de Lisboa, 1649-004 Lisboa, Portugal; monofre@fmh.ulisboa.pt; 3 ISAMB, Universidade de Lisboa, 1649-004 Lisboa, Portugal; amarques@fmh.ulisboa.pt (A.M.); mperalta@fmh.ulisboa.pt (M.P.); 4 CIPER, Faculdade de Motricidade Humana, Universidade de Lisboa, 1649-004 Lisboa, Portugal; duarteneto@campus.ul.pt; 5 Sports Studies and Physical Education Programme, School of Education, University College Cork, T12 YN60 Cork, Ireland; joao.costa@ucc.ie

**Keywords:** physical education, school, young people, survey, comparative research

## Abstract

Given the need for comparative research on the participation of adolescents in physical education (PE) classes as a privileged space for physical activity (PA) promotion, this study sought to estimate, analyse and compare the prevalence of participation in PE classes among adolescents from 54 countries and to examine sex, age, country income and world regions disparities. Data from the Global Students Health Survey (2010–2015) were used, comprising 170,347 adolescents (90,305 girls, aged 13–17 years) from nationally representative samples of 54 countries—of which 7 are low-income, 23 lower-middle-income, 14 upper-middle-income and 10 high-income—and six world regions. The weighted percentages of adolescents participating in PE classes (never, 1–2 days/week, 3–4 days/week, 5 or more days/week) were estimated along 95% confidence intervals and compared across sex, age, country income, region, and country. Most adolescents reported to participate in PE on 1–2 days/week (55.2%), but almost 20% of adolescents reported never participating in PE. Girls, compared to boys, presented a lower prevalence for participating ≥5 days/week (girls 16.8%, boys 20.0%). The prevalence of participating in PE on ≥3 days/week was higher among adolescents aged 13–14 years when compared to adolescents aged 15–17 years (boys: 30.9% vs. 24.6%; girls: 26.1% vs. 18.2%). Concerning the countries’ income, the prevalence of never participating in PE was higher in high-income countries, and participating on ≥3 days/week was higher in low-income countries, but further research is recommended. The findings suggest that national, regional and worldwide data highlight the importance of improving participation in PE, particularly for girls and older adolescents. An improved and continued monitoring of PE policies and their actual implementation is needed worldwide.

## 1. Introduction

Worldwide, the estimated global prevalence of adolescents practicing sufficient physical activity (PA) is low [1]. Given this scenario, the role of schools and physical education (PE) in promoting physically active and healthy lifestyles has been increasingly recognized as an essential component of the endeavours to develop a more active society, both in policy [2,3,4,5,6] and in research [7,8,9,10,11]. Several reasons have been advanced as to why schools and PE are of importance in promoting PA. The fact that school-based approaches can be cost-effective in promoting PA [8,12] and that most children and adolescents attend school are some of the main reasons [4,13]. Furthermore, for many children and adolescents, particularly those from disadvantaged backgrounds, PE may be the only setting where they engage in good-quality and meaningful PA experiences guided by a qualified specialist in the PE curriculum who provides pedagogically aligned lessons [4,14,15,16]. Additionally, there is evidence suggesting that PE can be an effective means to promote PA inside and outside of school [7,11,17,18]. Thus, participation in PE classes can be considered an important context for the promotion of PA in youth and have a role in reducing inequalities in the access to PA opportunities.

Despite the importance of PE in promoting PA, health and other socio-educational outcomes [18,19,20], PE still faces many challenges [21,22,23]. Some of these challenges are low subject status, insufficient time and reduced number of weekly PE lessons, and policy implementation gaps [4,13,22,24]. These challenges may have a direct impact on the number of opportunities students have to attend and participate in PE classes and, therefore, may be a threat to the effectiveness of PE in promoting active and healthy lifestyles. For this study, and in line with other studies [9,11,25], we are referring to the notion of PE participation as used by the underpinning research tool [26] which asks the students “During this school year, on how many days did you go to physical education (PE) class each week?”.

Considering the above-identified PE-related benefits and challenges, monitoring participation in PE classes is of importance, but research in this topic is mainly focused on high-income countries [4,27], demanding more updated information on low- and middle-income countries (LMIC) from different world regions [11,13,25]. Previous studies using different sources of information (e.g., experts, analyses of official documents) have found barriers to the access to PE for girls and older adolescents, particularly in some regions of the world and in LMIC [13,24,28]. However, those studies did not consider nationally representative samples of adolescents. Understanding the distribution of ‘PE participation’, stratified by sex and age, is needed and may allow further tailored actions in each region and country [29]. Therefore, and by using a pooled analysis of cross-sectional surveys, the present study aimed to estimate, analyse and compare the distribution of the prevalence of participation in PE classes among 13–17-year-old adolescents from 54 countries and additionally examine sex, age, country income and world regions disparities.

## 2. Materials and Methods

### 2.1. Data Source and Participants

Public data from the Global School-Based Student Health Survey (GSHS) were used (http://www.who.int/chp/gshs/datasets/en/). GSHS is a school-based and cross-sectional survey, aiming to provide data on health behaviours and protective factors of adolescents aged 13–17 years from several countries worldwide. The GSHS was developed by the WHO and the Centres for Disease Control and Prevention (CDC) in collaboration with the United Nations Children’s Fund, the United Nations Educational, Scientific and Cultural Organization (UNESCO) and The Joint United Nations Programme on HIV/AIDS. Country participation was voluntary. In each country, the GSHS used a common school-based methodology and a standardized two-stage sample selection to produce representative data of adolescent students [30]. Compared to other comparative approaches (e.g., Hardman, 2008, UNESCO, 2014), the GSHS provides a rich and different perspective, as it comes directly from the students on the number of days per week of PE classes.

From publicly available data collected between 2010 and 2015, we selected all nationally representative data sets that included the variables (PE participation, sex and age) and the information (weight, stratum and primary sampling unit) required for the analyses. The initial sample was 198,164 adolescents from 54 countries. As the survey was primarily designed for adolescents aged 13–17 years, students aged 11, 12 and 18 years were removed from the study sample (*n* = 19,982). Students with missing data for age (*n* = 1571), sex (*n* = 1125) and participation in PE classes (*n* = 5228) were also excluded from the analysis. The final sample comprised 170,347 adolescents (80,042 boys) aged 13–17 years from 54 countries, of which 7 are low-income, 23 lower-middle-income, 14 upper-middle-income, and 10 high-income (World Bank Classification, 2010–2015 data, https://www.worldbank.org) (see Supplementary Table S1).

### 2.2. Instrument

A self-administered questionnaire containing the GSHS core modules [26] allowed obtaining the data. The questionnaire was completed during one regular class period. Student participation was voluntary and private. Informed consent was previously obtained from schools’ representatives, parents and students. All procedures were in accordance with the ethical standards of the 1964 Helsinki declaration and its later amendments. 

As for participation in PE classes, the students were asked: “During this school year, on how many days did you go to physical education class each week?”. The response options were: (a) 0 days, (b) 1 day, (c) 2 days, (d) 3 days, (e) 4 days, (f) 5 or more days. PE participation has been recoded differently across studies, depending on each study purpose. For example, it has been recoded such as having PE classes on at least 1 day/week [9,27]; on ≥3 days/week and ≥5 days/week [25]; as the mean number days/week [27]; as the number of days/week (0, 1, 2, 3, 4, 5) [27,29]; and as never, 1–2 days/week and ≥3 days/week [11]. Similar to the last study, in the present work, PE participation was recoded into four categories: never, 1–2 days/week, 3–4 days/week and ≥5 days/week. Aligned with our study purpose, this discriminative approach allows obtaining a detailed understanding of the distribution of how many days each week did the students go to PE, which is important for tailored actions. Adolescents also reported their sex (male or female) and age (years). Based on CDC child developmental milestones [31], age was recoded into two categories: 13–14 years and 15–17 years.

### 2.3. Data Analysis

The GSHS survey employs a two-stage complex sampling design [30]. Thus, to appropriately represent the weighting process and the two-stage sample design, all analyses accounted for the “weight”, “stratum” and “primary sampling unit” (PSU) variables, as recommended [30]. The “weight” allows GSHS results to be generalized to the entire population of adolescent students. The “stratum” and “PSU” reflect, respectively, the first level (schools) and the second level (classrooms) of the GSHS sample selection process. Further details on the complex sample procedures of the GSHS can be found elsewhere [32].

To estimate the participation in PE classes (never, 1–2 days/week, 3–4 days/week, ≥5 days/week), data were stratified by sex, age, world regions, country income classification and countries. Descriptive data are presented as weighted percentages. The weighted percentages of adolescents in each level of participation in PE classes was calculated along with a 95% confidence interval. All statistical analyses were performed using the complex samples menu of the IBM SPSS Statistics version 25.0 (IBM, New York, NY, USA)

## 3. Results

Table 1 presents the sample characteristics and distribution according to age, country income and PE participation. Most students reported to participate in PE classes on 1–2 days/week (55.3, 95% CI: 53.1, 57.4). However, 18.1% (95% CI: 16.8, 19.4) reported no participation in PE, and 18.5% (95% CI: 17.5, 19.9) reported participation on ≥5 days/week. The percentage of boys who reported to participate in PE classes on 1–2 days/week was 53.9% (95% CI: 51.6, 56.2), and for girls was 56.7% (95% CI: 54.2, 59.1). Girls presented a lower prevalence for participating in PE classes on ≥5 days/week (16.8, 95% CI: 15.7, 17.9 vs. 20.0, 95% CI: 18.7, 21.4).

In Table 2, the prevalence of PE participation stratified by age groups is presented. The prevalence of adolescents who participated 1–2 days/week in PE classes tended to increase for the older age groups compared to the younger ones for boys (from 51.3, 95% CI: 48.5, 54.1 to 56.2, 95% CI: 53.4, 59.0) and girls (from 52.1, 95% CI: 49.4, 54.7 to 61.0, 95% CI: 58.0, 63.9). However, this increase was accompanied by a decrease with age in the prevalence of adolescents who revealed to participate in PE classes on 5 or more days/week, both for boys (21.8% to 18.5%) and for girls (19.4% to 14.3%).

The distribution of adolescents’ participation in PE according to the country income is presented in Table 3. For boys and girls, the highest prevalence of adolescents who said never participating in PE was found in high-income countries (boys: 23.4%, 95% CI: 20.1, 27.0; girls: 24.7%, 95% CI: 21.1, 28.7). Conversely, low-income countries had a higher percentage of adolescents reporting to participate ≥3 days/week in PE (boys: 44.0%, girls: 36.4%). A decreasing trend in PE participation on ≥3 days/week was observable from low- to lower-middle (boys: 26.3%, girls: 23.2%) and upper-middle-income countries (boys: 21.6%, girls: 17.6%). The percentage of boys and girls who participated in PE on 1–2 days/week increased from low-income (boys: 38.4, 95% CI: 34.1, 42.8; girls: 40.5, 95% CI: 37.3, 43.7) to upper-middle-income countries (boys: 64.9, 95% CI: 63.1, 66.7; girls: 71.2, 95% CI: 69.0, 73.3).

The percentage of participation in PE classes for boys and girls across regions is presented in Table 4.

The highest percentage of adolescents who reported to never participate in PE classes was observed in boys from Sub-Saharan Africa (28.7, 95% CI: 25.6, 32.1) and in girls from Central Asia, the Middle East and North Africa (38.8, 95% CI: 33.6, 44.1). The region showing the highest prevalence for 1–2 days/week of PE was East and Southeast Asia for both boys (64.2, 95% CI: 60.9, 67.4) and girls (69.1, 95% CI: 65.8, 72.1). Boys and girls from South Asia (that only included two countries) had the highest prevalence in ≥3 days/week of PE participation (50.2% and 46.0%). Considering the other regions (that included between 8 to 14 countries each), the higher percentages of boys and girls reporting to participate in PE classes on ≥3 days/week were observable in Sub-Saharan Africa (32.5%) and Oceania (29.7%), respectively.

**Figure 1:**
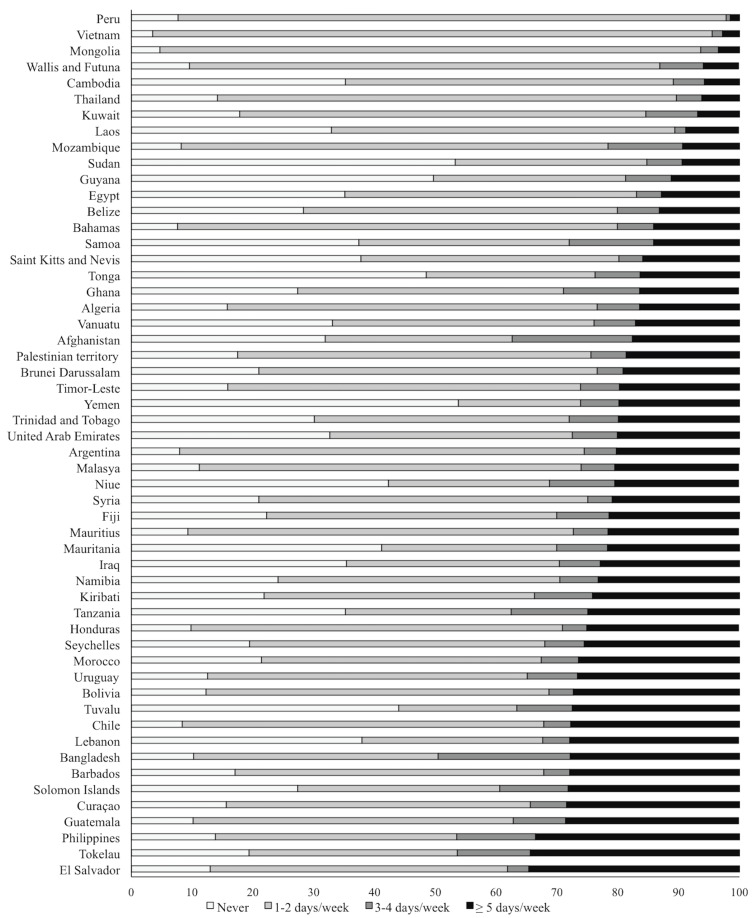
Participation in physical education classes by countries.

Figure 1 (and Supplementary Table S2) presents the distribution of participation in PE classes (days/week) per country. The countries reporting higher percentages of the prevalence of no participation in PE were Yemen in Central Asia (53.8, 95% CI: 46.4, 61.0), Sudan in Sub-Saharan Africa (53.3, 95% CI: 43.6, 62.8), Guyana in Sub-Saharan Africa (49.7, 95% CI: 43.0, 56.4), Tonga (48.9, 95% CI: 44.8, 52.2) and Tuvalu (44.0, 95% CI: 44.0, 44.0), both in Oceania, Afghanistan in South Asia (31.9, 95% CI: 25.5, 39.2) and Cambodia in East and Southeast Asia (35.2. 95% CI: 30.7, 39.9).

Countries that presented the highest percentage of adolescents reporting to participate in PE classes on 5 or more days/week were Tokelau in Oceania (34.5, 95% CI: 19.7, 52.9), the Philippines in East and Southeast Asia (33.5, 95% CI: 30.3, 36.8), Lebanon in Central Asia (27.8, 95% CI: 24.4, 31.5), Bangladesh in South Asia (27.8, 95% CI: 24.1, 31.8), Seychelles in Sub-Saharan Africa (25.6, 95% CI: 23.0, 28.3) and El Salvador (34.6, 95% CI: 31.3, 38.1), Guatemala (28.5, 95% CI: 22.8, 35.0) and the Curaçao (28.4, 95% CI: 25.4, 31.6) all in Latin America and the Caribbean. 

## 4. Discussion

This study summarized and compared nationally representative data concerning participation in PE classes among 170,354 adolescents, which adds to previous cross-national research on participation in PE classes [4,25,29] by including a greater number of countries from different income levels and from six world regions, as well as by quantifying sex, age, country income and region disparities. Regardless of the argument for PE being more philosophical, pedagogical or political, no foreseen present or future of PE [21,22,33] can be achieved without knowing the PE participation time where students strive to achieve psychosocioeducational outcomes and health benefits through their educational programmes. From comparing and analysing participation in PE, all stakeholders can engage in deep and meaningful discussions about the reasons and possible solutions for present and recurrent challenges, which is our aim in this discussion.

Almost 20% of adolescents reported not to participate in PE during the school year, suggesting persistent gaps between PE policies and implementation [13,28]. Conversely, almost 20% of adolescents answered to participate in PE classes on ≥5 days per week. These data suggest that there is still a lot of space for improvement in PE participation. Governments can provide leadership by requiring and supporting schools to provide good-quality, daily PE and by improving their national surveillance of PE quantity and quality [3,4,5,10].

Daily PE and/or PA sessions in schools have been recommended in Europe [3] and the United States [2,34] and are being implemented in some countries or districts [4,27]. On the one hand, this is important because higher PE participation aligned with good-quality experiences can lead to more PA practice [11,35], as well as to psychosocial, cognitive and health benefits [18,19,20,36]. On the other hand, more of the same “traditional” PE (i.e., sports-based techniques) does not seem to be the long-term solution for PE to overcome the continuous challenges it has been facing (Kirk, 2010). Thus, it is important to highlight that the focus should also be placed on the internal professional responsibility to change pedagogies and practices and make PE more dynamic, inclusive and effective in promoting active lifestyles [4,22,23]. The development observed around the health-based PE is encouraging in this respect [37,38]. Continuous efforts and research with higher quality evidence for this and for the impact of PE on lifelong engagement in PA are needed and encouraged [37,38,39].

Boys reported higher participation in PE lessons on ≥3 days/week when compared to girls. Contrariwise, some studies provide evidence that, in some countries, equal participation opportunities to boys and girls are being provided [25,29]. However, and despite some noted improvement in gender-related inclusion policies and practices [13], other studies highlight lower PE participation rates of girls in some countries and regions [4,13,27]. In line with these studies, our results also suggest that some barriers to the equal provision of PE for girls may remain, namely, in accessing more than two PE classes per week. The reasons for these differences may vary within and across regions and countries and can be related to social barriers, religion–cultural dispositions, parental discouragement, and PE socio-educational status and resources [13,40]. 

Considering these results of our study, girls may be in greater need than boys of intervention to have equal access to participate in PE [4,5,13,40]. Importantly, based on this study, each country and region need to further explore the reasons for these results as well as the types of barriers in place and set actions in order to reduce the number of adolescents who reported never having PE classes (boys: 16.9%, girls: 19.3%). When in PE classes, instead of pre-dominant experiences related to competitive sports [4,13,40], evidence also suggests that girls should be provided with more personally meaningful and socially relevant experiences that help them to develop the skills, motivation and physical competence to become and remain physically active [14,41]. Getting more girls more active is of importance, and schools and PE contributions are fundamental in order to help to overcome this global challenge [1,6].

Comparing to adolescents aged 13–14 years, a lower percentage of older adolescents mentioned participating in PE classes on ≥3 days per week. Issues of PE lessons’ frequency and time allocation during the schooling years are complex due to localised control of curricula and practices of offering options to the students [4,13]. While, globally, there seems to be a similar average time allocated to primary and secondary school levels to PE, both regional and national important variations have been identified [13,27,42]. Thus, since PA tends to decline from childhood to adolescence [43], assuring the involvement of older students in the same number or more PE classes as their age increases might be an important strategy to promote PA and health and socio-educational benefits. In this regard, educational authorities can play a critical role in defining a mandatory minimum of minutes and number of PE lessons per week throughout the schooling years. Internally, PE professionals should also endeavour efforts to revert the tendency for students’ attitudes towards PE decrease with age [44]. In this regard, future research should identify factors that decrease or stabilize PE attitude with age [44].

The higher prevalence of adolescents reporting “never” participating in PE was found in high-income countries, and reporting ≥3 days per week was observed in low-income countries. Additionally, a decreasing trend of the prevalence of participating in PE on ≥3 days per week was detected from low- to upper-middle-income countries. These results seem to be contradictory, since in high-income countries policies and the provision of built environments tend to facilitate involvement in PE/PA opportunities in schools [13,45]. Possible explanations for divergences are complex and might be related to the multiple income indicators used, the number and types of countries integrated into each income level and the regions of those countries. Additionally, it may not reflect further PA school-based opportunities, like clubs and school sports as co-curricular offers. Nevertheless, other studies have found no relation or inconsistent associations between PE frequency and country-level income indicators [25,29]. Thus, these results should be interpreted with caution, and further research is needed to explore the relationship between PE participation and different income-related indicators.

The higher percentage of adolescents “never” participating in PE classes was observed for boys and girls from Sub-Saharan Africa regions, as well as for girls from Central Asia and the Middle East and North Africa. These results are similar to UNESCO’s [13] findings, indicating that Africa and Middle East regions had higher percentages of countries where PE was not being implemented in accordance with mandatory obligations. South Asia (only with two countries), Oceania and Sub-Saharan Africa were the regions with a higher percentage of adolescents reporting to participate in daily PE. Reasons such as PE mandatory minutes, cancellations, facilities and the number of qualified teachers might help to explain those variations across and within regions and countries [13,29]. Concurrently, these numbers need to be framed in the context of the more general educational access opportunities according to social and religious norms. Understanding regional and national differences in PE participation can also facilitate the identification of priorities and the design of tailored actions across the region, country and local contexts.

Since PE policy implementation remains inconsistent [13], advancing implementation and continued surveillance of quality PE should be prioritized at a regional and national level. This might help to improve the frequency of PE classes available and students’ participation, particularly amongst girls and older adolescents, and consequently contribute to their PA and physical literacy [33,46]. In addition to PE quantity, indicators of quality of PE should be considered and monitored (e.g., teacher behaviours, learning outcomes) [5]. The European Physical Education Observatory (EUPEO) provides an important action in this direction [47] by offering an evaluation platform that captures the alignment between policy, practices and experiences in PE according to the UNESCO Quality PE framework [5]. Another important example in this direction is the innovative conceptual assessment framework proposed for quality PE and health-optimizing physical education that serve health and educative goals [15], a theme that seems to still divide the PE community [21,37,38].

Some limitations of this study should be acknowledged. The questions that compose the core models of the GSHS questionnaire draw from the Youth Risk Behaviour Survey [27,48]. No information on the validity and reliability of the instrument/questions was available for the included countries. However, only a single simple question was used to assess the frequency of PE. Also, the used question about PE does not discriminate the reasons for not participating in PE classes and does not indicate if attending PE classes reflects actual participation, for example, in learning tasks and moderate to vigorous physical activity. Future research can consider these issues, as well as include questions related to PE lesson time and PE quality, and use different approaches to collect and triangulate data. Data on PE participation was collected for each country in one year between 2010 and 2015. This might affect comparisons, and future studies should compare only surveys from the same year. In addition to the external validity, the standardized methodology and the high response rates to the questionnaire are the main strengths of the present study. By relying on large and nationally representative samples of adolescents, mainly from LMIC of different world regions, this study is unique and adds to previous cross-national research on participation in PE during adolescence [25,29].

## 5. Conclusions

National, regional and worldwide data highlight the importance of further improving the participation in PE lessons for all adolescents, particularly for girls and older adolescents. Current data have shown that only about 20% of adolescents reported participating in daily PE, as many international authorities have been recommending. Additionally, about 20% of adolescents reported never attending PE. Identifying the factors related to this phenomenon is needed and may allow regional, national and international authorities to establish priority actions and policies against reduced participation in PE classes. The results of our study also suggest that there is still too much of a gap between PE policies and their actual implementation, as well as that barriers to the equal provision of PE frequency for girls remain. This situation needs to be reversed. Thus, an improved and continued monitoring of PE policies and their actual implementation is needed worldwide, as are innovation and empirical evidence on new PE pedagogies that help to make children and adolescents more active and healthier.

## Figures and Tables

**Table 1:** Adolescents’ characteristics stratified by sex.

	Total			Boys			Girls		
Adolescents’ Characteristics	Sample	Weighted Sample		Sample	Weighted Sample		Sample	Weighted Sample	
	*n*	*n*	% (95% CI)	*n*	*n*	% (95% CI)	*n*	*n*	% (95% CI)
**Age**									
13–14 years	81,591	21,787,465.554	48.0 (45.7, 50.4)	37,589	11,075,629.553	47.5 (44.8, 50.2)	44,003	10,711,887.770	48.6 (46.1, 51.1)
15–17 years	88,756	23,564,472.292	52.0 (49.6, 54.3)	42,456	12,237,987.930	52.5 (49.8, 55.2)	46,303	11,326,780.925	51.4 (48.9, 53.9)
**Income classification**									
High	15,296	45,351,937.846	0.8 (0.7, 1.0)	7144	181,157.000	0.8 (0.6, 1.0)	8152	203,637.528	0.9 (0.7, 1.2)
Upper-middle	79,840	10,487,049.268	23.1 (21.5, 24.9)	37,889	5,103,499.635	21.9 (20.0, 23.9)	41,951	5,383,549.634	24.4 (22.6, 26.3)
Lower-middle	60,637	25,715,440.834	56.7 (53.9, 59.5)	28,350	12,858,975.291	55.2 (51.6, 58.7)	32,287	12,856,465.543	58.3 (55.6, 61.0)
Low	14,574	8,764,653.216	19.3 (16.0, 23.2)	6659	5,169,688.995	22.2 (17.9, 27.7)	7915	3,594,964.222	16.3 (13.4, 19.7)
**Physical education**									
Never	31,371	8,200,192.066	18.1 (16.8, 19.4)	14,486	7,558,289.838	16.9 (15.6, 18.2)	16,885	4,260,523.860	19.3 (17.5, 21.3)
1–2 days/week	94,839	25,057,929.299	55.3 (53.1, 57.4)	43,391	12,567,385.018	53.9 (51.6, 56.2)	51,450	12,490,661.791	56.7 (54.2, 59.1)
3–4 days/week	11,324	3,725,650.092	8.2 (7.5, 9.0)	5601	2,137,064.064	9.2 (8.1, 10.4)	5723	1,588,586.027	7.2 (6.5, 8.0)
≥5 days/week	32,813	8,368,166.390	18.5 (17.5, 19.5)	16,566	4,669,321.142	20.0 (18.7, 21.4)	16,247	3,698,845.248	16.8 (15.7, 17.9)
**Total**	170,348	45,352,197.784	100	80,042	23,313,320.920	100	90,305	22,038,616.927	100

**Table 2:** Participation in physical education classes according to age, stratified by sex.

Participation in PE	13–14 Years	15–17 Years
**Boys**	**Weighted % (95% CI)**	**Weighted % (95% CI)**
Never	17.6 (15.8, 19.5)	16.3 (15.0, 17.7)
1–2 days/week	51.3 (48.5, 54.1)	56.2 (53.4, 59.0)
3–4 days/week	9.3 (7.9, 11.0)	9.0 (7.9, 10.3)
≥5 days/week	21.8 (20.2, 23.4)	18.5 (17.0, 20.0)
**Girls**	**Weighted % (95% CI)**	**Weighted % (95% CI)**
Never	20.7 (18.3, 23.2)	18.1 (16.0, 20.3)
1–2 days/week	52.1 (49.4, 54.7)	61.0 (58.0, 63.9)
3–4 days/week	7.9 (6.9, 8.9)	6.6 (5.8, 7.5)
≥5 days/week	19.4 (18.0, 20.8)	14.3 (13.2, 15.6)

**Table 3:** Participation in physical education classes according to country income classification, stratified by sex.

	Income Classification			
Participation in PE	Low	Lower-Middle	Upper-Middle	High
**Boys**	**Weighted % (95% CI)**	**Weighted % (95% CI)**	**Weighted % (95% CI)**	**Weighted % (95% CI)**
Never	17.6 (14.8, 20.9)	17.8 (16.0, 19.9)	13.5 (12.4, 14.8)	23.4 (20.1, 27.0)
1–2 days/week	38.4 (34.1, 42.8)	55.8 (52.7, 58.9)	64.9 (63.1, 66.7)	52.1 (47.4, 56.9)
3–4 days/week	18.8 (15.5, 22.6)	6.8 (6.1, 7.7)	5.3 (4.6, 6.1)	8.9 (7.5, 10.6)
≥5 days/week	25.2 (22.0, 28.8)	19.5 (17.8, 21.3)	16.3 (15.0, 17.6)	15.6 (13.1, 18.4)
**Girls**	**Weighted % (95% CI)**	**Weighted % (95% CI)**	**Weighted % (95% CI)**	**Weighted % (95% CI)**
Never	23.2 (20.5, 26.2)	21.6 (18.9, 24.6)	11.1 (9.5, 12.8)	24.7 (21.1, 28.7)
1–2 days/week	40.5 (37.3, 43.7)	55.2 (51.5, 58.8)	71.2 (69.0, 73.3)	54.7 (49.7, 59.7)
3–4 days/week	14.8 (12.3, 17.6)	6.4 (5.5, 7.3)	4.2 (3.7, 4.7)	6.5 (5.4, 7.7)
≥5 days/week	21.6 (19.2, 24.1)	16.8 (15.1, 18.7)	13.6 (12.3, 14.9)	14.1 (11.8, 16.8)

**Table 4:** Participation in physical education classes according to regions, stratified by sex.

Participation in PE	Central Asia, Middle East, and North Africa	East and Southeast Asia	Oceania	Sub-Saharan Africa	South Asia	Latin America and Caribbean
**Boys**	**Weighted %** **(95% CI)**	**Weighted %** **(95% CI)**	**Weighted %** **(95% CI)**	**Weighted** **% (95% CI)**	**Weighted %** **(95% CI)**	**Weighted %** **(95% CI)**
Never	24.2 (20.9, 27.8)	12.9 (11.6, 14.3)	27.1 (24.3, 30.0)	28.7 (25.6, 32.1)	11.4 (8.0, 16.0)	10.3 (9.4, 11.3)
1–2 days/week	48.7 (44.5, 52.9)	64.2 (60.9, 67.4)	41.5 (38.0, 45.1)	38.7 (34.8, 42.7)	38.4 (32.1, 45.1)	63.8 (61.4, 66.2)
3–4 days/week	6.2 (5.4, 7.0)	6.7 (5.7, 7.8)	9.6 (7.9, 11.5)	11.3 (9.8, 13.0)	23.0 (18.2, 28.6)	4.6 (3.6, 5.9)
≥5 days/week	20.9 (18.7, 23.3)	16.2 (14.4, 18.2)	21.9 (19.7, 24.3)	21.3 (18.1, 24.9)	27.2 (22.6, 32.4)	21.2 (19.3, 23.2)
**Girls**	**Weighted %** **(95% CI)**	**Weighted %** **(95% CI)**	**Weighted %** **(95% CI)**	**Weighted %** **(95% CI)**	**Weighted %** **(95% CI)**	**Weighted %** **(95% CI)**
Never	38.8 (33.6, 44.1)	9.2 (8.1, 10.5)	27.2 (23.5, 31.1)	35.8 (32.2, 39.6)	11.9 (8.8, 15.9)	9.3 (8.2, 10.4)
1–2 days/week	42.2 (37.4, 47.2)	69.1 (65.8, 72.1)	43.1 (39.4, 46.9)	36.6 (33.5, 39.7)	42.0 (36.1, 48.1)	67.2 (64.9, 69.5)
3–4 days/week	4.7 (4.0, 5.5)	6.2 (5.2, 7.3)	8.9 (7.8, 10.1)	11.4 (9.9, 13.0)	18.9 (14.4, 24.5)	4.0 (3.2, 4.8)
≥5 days/week	14.4 (12.3, 16.7)	15.5 (13.7, 17.6)	20.8 (18.5, 23.3)	16.2 (14.1, 18.6)	27.1 (23.9, 30.7)	19.6 (17.8, 21.5)

## Supplementary Materials

The following are available online at https://www.mdpi.com/1660-4601/17/15/5579/s1, Table S1: Survey year and sample size for countries that participated in the Global School-Based Health Survey, 2010–2015., Table S2: Participation in physical education classes by countries.
