# Supplementary material for: A Comparative Study of Participation in Physical Education Classes among 170,347 Adolescents from 54 Low-, Middle-, and High-Income Countries
Source: Int J Environ Res Public Health. 2020 Aug 2;17(15):5579. doi: 10.3390/ijerph17155579 (PMC7432845; doi:10.3390/ijerph17155579)
Supplement: Supplementary file 1 [file ijerph-17-05579-s001.pdf]

Supplementary material

Table S1. Survey year and sample size for countries that participated in the Global School-Based Health Survey, 2010–2015.

Country by Region	Income Classification	Survey Year	Overall Response Rate (%) *	Final Sample **	Weighted % of Girls in Final Sample (95% CI)	Mean Age of Final Sample (95% CI)
Central Asia, Middle East, and north Africa						
Algeria	Lower-upper	2011	98	3765	46.3 (30.1, 63.3)	14.5 (14.4,14.6)
Egypt	Lower-middle	2011	85	2041	50.1 (39.3, 61.0)	13.8 (13.7, 13.9)
Iraq	Lower-middle	2012	88	1811	42.5 (29.6, 56.6)	14.6 (14.4, 14.7)
Kuwait	High	2015	98	3041	49.6 (32.4, 66.8)	15.1 (14.7, 15.5)
Lebanon	Lower-upper	2011	87	1907	52.6 (48.0, 57.2)	14.2 (14.1, 14.3)
Mongolia	Lower-middle	2013	88	4466	51.3 (49.5, 53.2)	14.8 (14.7, 14.9)
Morocco	Lower-middle	2010	92	2346	42.8 (39.1, 46.6)	14.3 (14.2, 14.4)
Palestinian territory	Lower-middle	2010	94	3835	48.3 (32.4, 64.6)	14.1 (13.9, 14.1)
Syria	Lower-middle	2010	97	2551	48.3 (38.4, 58.4)	13.9 (13.8, 14.1)
United Arab Emirates	High	2010	91	2361	58.7 (44.3, 71.8)	14.3 (14.1, 14.4)
Yemen	Lower-middle	2014	75	1993	46.3 (34.7, 58.4)	14.9 (14.7, 15.1)
East and Southeast Asia						
Brunei Darussalam	High	2014	65	2361	50.1 (45.8, 54.5)	14.6 (14.5, 14.8)
Cambodia	Low	2013	85	2807	49.7 (47.6, 51.8)	15.0 (14.8,15.2)
Laos	Lower-middle	2015	70	3587	47.0 (44.6, 49.4)	15.8 (15.6, 16.0)
Malaysia	Lower-upper	2012	99	24,921	50% (48.1, 51.9)	14.9 (14.8, 15.0)
Philippines	Lower-middle	2015	79	7738	51.2 (47.9, 54.5)	14.6 (14.5, 14.7)
Thailand	Lower-upper	2015	89	4881	52.9 (48.7, 57.1)	14.8 (14.6, 14.9)
Timor-Leste	Lower-middle	2015	79	2669	52.1 (49.8, 54.3)	15.3 (15.2, 15.8)
Vietnam	Lower-middle	2013	96	3005	53.6 (48.0, 59.1)	15.5 (15.3, 15.7)
Latin America and Caribbean						
Argentina	Lower-upper	2012	71	25,651	52.5 (50.2, 54.8)	14.5 (14.4, 14.5)
Bahamas	High	2013	78	1058	51.4 (47.4, 55.4)	13.9 (13.6, 14.0)
Barbados	High	2011	73	1548	50.9 (45.9, 55.9)	14.3 (14.1, 14.5)
Belize	Lower-middle	2011	88	1564	51.2 (46.6, 55.7)	14.4 (14.2, 14.6)
Bolivia	Lower-middle	2012	88	3249	49.1 (46.7, 51.5)	14.5 (14.4, 14.7)
Chile	Lower-upper	2013	60	1598	51.7 (45.8, 57.6)	15.0 (14.8, 15.0)
Curaçao	High	2015	83	1884	51.3 (46.7, 56.0)	15.1 (14.9, 15.4)
El Salvador	Lower-middle	2013	88	1767	48.2 (43.7, 52.7)	14.3 (14.2, 14.4)
Guatemala	Lower-middle	2015	82	3787	47.2 (43.3, 51.1)	14.4 (14.2, 14.7)

Guyana	Lower-middle	2010	76	2277	51.5 (48.0, 55.0)	14.4 (14.2, 14.5)
Honduras	Lower-middle	2012	79	1440	52.3 (48.3, 56.3)	14.2 (14.1, 14.3)
Peru	Lower-upper	2010	85	2802	49.5 (43.1, 56.0)	14.5 (14.4, 14.6)
Saint Kitts and Nevis	Lower-upper	2011	70	1648	49.5 (49.5, 49.5)	14.5 (14.5, 14.5)
Trinidad and Tobago	High	2011	90	2037	51.7 (41.4, 61.8)	14.3 (14.1, 14.4)
Uruguay	Lower-upper	2012	77	3344	54.5 (52.7, 56.3)	14.4 (14.3, 14.5)
Oceania						
Fiji	Lower-upper	2010		1610	50.8 (45.0, 56.6)	14.3 (13.9, 14.6)
Kiribati	Lower-middle	2011	85	1483	53.4 (49.7, 57.0)	14.6 (14.2, 14.5)
Niue	High	2010	99	102	39.2 (39.2, 39.2)	15.1 (15.1, 15.1)
Samoa	Lower-middle	2011	92	1984	52.7 (49.4, 56.0)	14.2 (14.1, 14.3)
Solomon Islands	Low	2011	85	1214	45.5 (41.2, 49.9)	14.8 (14.6, 14.9)
Tokelau	High	2014	71	85	46.8 (30.6, 63.8)	14.6 (14.2, 14.9)
Tonga	Lower-middle	2010	80	1998	48.4 (43.7, 53.1)	14.4 (14.3, 14.6)
Tuvalu	Lower-upper	2013	90	673	51.9 (51.9, 51.9)	14.6 (14.6, 14.6)
Vanuatu	Lower-middle	2011	72	845	51.1 (47.0, 55.1)	14.3 (14.0, 14.5)
Wallis and Futuna	High	2015	82	819	53.6 (48.0, 59.1)	15.0 (14.8, 15.3)
South Asia						
Afghanistan	Low	2014	79	1889	46.3 (30.1, 63.3)	15.0 (14.8, 15.1)
Bangladesh	Low	2014	91	2757	33.9 (26.5, 42.2)	14.2 (14.0, 14.4)
Sub-Saharan Africa						
Ghana	Lower-middle	2012	82	2142	49.5 (46.2, 52.9)	15.2 (14.9, 15.3)
Mauritania	Low	2010	82	1810	45.9 (39.5, 52.4)	15.0 (14.8, 15.1)
Mauritius	Lower-upper	2011	82	1957	51.5 (34.6, 68.1)	14.1 (14.0, 14.2)
Mozambique	Low	2015	80	1241	47.5 (43.3, 51.8)	15.1 (14.8, 15.4)
Namibia	Lower-upper	2013	89	3218	55.4 (53.3, 57.5)	15.2 (15.0, 15.4)
Seychelles	Lower-upper	2015	93	1865	51.4 (48.5, 54.4)	14.5 (14.3, 14.7)
Sudan	Lower-middle	2012	77	2059	46.8 (30.2, 64.2)	14.8 (14.6, 15.0)
Tanzania	Low	2014	87	2856	50.1 (46.9, 53.3)	14.5 (14.3, 14.8)
**Total**				**170,347**	**48.6 (46.9, 50.3)**	**14.7 (14.6,14.8)**

* Overall response rate refers to the entire sample including those students outside the targeted age range of 13 to 17 years. Source: Uddin, R., Lee, E., Khand, S., Tremblay, M., Khan, A. (2020). Clustering of lifestyle risk factors for non-communicable diseases in 304,779 adolescents from 89 countries: A global perspective. Preventive Medicine doi:10.1016/j.ypmed.2019.105955. ** Students aged 13–17 years with complete data on PE, sex, age for ‘weighting’ the analysis.

Table S2. Participation in physical education classes by countries.

Region	Country	Physical Education			
		Never	1–2 Days/Week	3–4 Days/Week	≥5 Days/Week
		Weighted % (95% CI)	Weighted % (95% CI)	Weighted % (95% CI)	Weighted % (95% CI)
South Asia	Afghanistan	31.9 (25.5, 39.2)	30.7 (26.1, 35.7)	19.8 (13.5, 28.1)	17.6 (13.5, 22.5)
Central Asia, Middle East, and north Africa	Algeria	15.8 (11.9, 20.7)	60.8 (54.4, 65.0)	7.0 (5.8, 8.5)	16.4 (14.0, 19.1)
Latin America and Caribbean	Argentina	8.0 (7.2, 9.0)	66.5 (64.6, 68.4)	5.3 (4.5., 6.3)	20.2 (18.9, 21.5)
Latin America and Caribbean	Bahamas	7.6 (5.9, 9.7)	72.3 (67.8, 76.3)	6.0 (4.4, 8.2)	14.1 (10.8, 18.4)
South Asia	Bangladesh	10.3 (7.8, 13.6)	40.2 (34.7, 46.0)	21.7 (17.5, 26.5)	27.8 (24.1, 31.8)
Latin America and Caribbean	Barbados	17.1 (13.2, 21.9)	50.7 (46.7, 54.6)	4.3 (3.4, 5.6)	27.9 (25.1, 30.9)
Latin America and Caribbean	Belize	28.3 (20.1, 38.2)	51.6 (44.1, 59.0)	6.9 (4.4, 10.6)	13.3 (10.6, 16.5)
Latin America and Caribbean	Bolivia	12.3 (10.8, 13.8)	56.4 (53.9, 58.9)	4.0 (3.3, 5.0)	27.3 (25.4, 29.4)
East and southeast Asia	Brunei Darussalam	21.0 (16.6, 26.2)	55.6 (51.3, 59.8)	4.3 (3.5, 5.2)	19.2 (17.3, 21.2)
East and southeast Asia	Cambodia	35.2 (30.7, 39.9)	53.9 (49.1, 58.6)	5.1 (4.2, 6.2)	5.9 (4.7, 7.3)
Latin America and Caribbean	Chile	8.4 (5.4, 13.0)	59.4 (55.3, 63.4)	4.5 (3.1, 6.4)	27.7 (24.5, 31.2)
Latin America and Caribbean	Curaçao	15.6 (13.1, 18.3)	50.0 (46.3, 53.8)	6.0 (4.9, 7.4)	28.4 (25.4, 31.6)
Central Asia, Middle East, and north Africa	Egypt	35.1 (27.5, 43.6)	48.0 (38.6, 57.5)	4.1 (2.9, 5.8)	12.8 (9.9, 16.3)
Latin America and Caribbean	El Salvador	13.0 (9.6, 17.4)	48.9 (44.5, 53.2)	3.5 (2.6, 4.9)	34.6 (31.3, 38.1)
Oceania	Fiji	22.3 (18.9, 26.2)	47.7 (43.6, 51.8)	8.6 (7.0, 10.5)	21.4 (18.8, 24.3)
Sub-Sharan Africa	Ghana	27.4 (22.9, 32.6)	43.7 (38.3, 49.3)	12.5 (9.8, 15.9)	16.3 (12.4, 21.2)
Latin America and Caribbean	Guatemala	10.2 (8.6, 12.2)	52.6 (44.3, 60.8)	8.6 (3.5, 19.7)	28.5 (22.8, 35.0)
Latin America and Caribbean	Guyana	49.7 (43.0, 56.4)	31.6 (26.3, 37.4)	7.5 (6.0, 9.2)	11.3 (9.7, 13.2)
Latin America and Caribbean	Honduras	9.8 (7.1, 13.5)	61.1 (56.9, 65.2)	4.0 (3.1, 5.1)	25.0 (22.3, 28.0)
Central Asia, Middle East, and north Africa	Iraq	35.4 (30.2, 41.1)	35.0 (31.1, 39.1)	6.7 (5.7, 7.8)	22.9 (20.1, 25.9)
Oceania	Kiribati	21.8 (17.9, 26.2)	44.5 (40.6, 48.4)	9.5 (8.1, 11.1)	24.3 (21.2, 27.6)
Central Asia, Middle East, and north Africa	Kuwait	17.8 (14.5, 21.7)	66.8 (61.8, 71.4)	8.5 (6.4, 11.3)	6.9 (5.2, 9.1)
East and southeast Asia	Laos	32.9 (27.0, 39.4)	56.5 (50.0, 62.8)	1.8 (1.3, 2.6)	8.7 (7.3, 10.4)
Central Asia, Middle East and north Africa	Lebanon	37.9 (31.8, 44.4)	29.8 (25.9, 34.1)	4.4 (3.4, 5.7)	27.8 (24.4, 31.5)
East and southeast Asia	Malaysia	11.2 (9.9, 12.6)	62.8 (61.2, 64.4)	5.5 (5.0, 6.1)	20.4 (19.4, 21.5)

Sub-Sharan Africa	Mauritania	41.2 (35.4, 47.2)	28.8 (24.9, 33.0)	8.3 (6.8, 10.2)	21.7 (18.4, 25.5)
Sub-Sharan Africa	Mauritius	9.3 (7.2, 11.9)	63.4 (59.6, 67.1)	5.7 (4.3, 7.6)	21.5 (18.9, 24.4)
Central Asia, Middle East, and north Africa	Mongolia	4.7 (378, 6.0)	88.9 (86.5, 90.9)	2,9 (1.8, 4.6)	3.5 (2.8, 4.3)
Central Asia, Middle East, and north Africa	Morocco	21.4 (18.2, 25.2)	46.0 (42.3, 49.7)	6.1 (4.9, 7.4)	26.5 (24.1, 29.0)
Sub-Sharan Africa	Mozambique	8.2 (5.1, 13.0)	70.2 (62.8, 76.6)	12.3 (8.5, 17.5)	9.3 (6.3, 13.5)
Sub-Sharan Africa	Namibia	24.1 (19.2, 29.6)	46.4 (42.4, 50.4)	6.3 (5.2, 7.7)	23.2 (20.1, 26.7)
Oceania	Niue	42.3 (42.3, 42.3)	26.5 (26.5, 26.5))	10.7 (10.7, 10.7)	20.4 (20.4, 20.4)
Central Asia, Middle East, and north Africa	Palestinian territory	17.5 (15.0, 20.4)	58.1 (55.1, 61.0)	5.8 (4.9, 6.9)	18.6 (16.7, 20.7)
Latin America and Caribbean	Peru	7.7 (6.4, 9.2)	90.1 (87.5, 92.2)	0.7 (0.4, 1.1)	1.5 (0.6, 4.0)
East and southeast Asia	Philippines	13.8 (12.0, 15.9)	39.7 (35.6, 43.9)	13.0 (11.2, 15.1)	33.5 (30.3, 36.8)
Latin America and Caribbean	Saint Kitts and Nevis	37.8 (37.8, 37.8)	42.4 (42.4, 42.4)	3.9 (3.9, 3.9)	15.9 (15.9, 15.9)
Oceania	Samoa	37.4 (34.9, 39.)	34.6 (30.8, 38.5)	13.9 (12.3, 15.7)	14.2 (11.8, 16.8)
Sub-Sharan Africa	Seychelles	19.5 (16.6, 22.7)	48.5 (45.6, 51.4)	6.5 (5.4, 7.7)	25.6 (23.0, 28.3)
Oceania	Solomon Islands	27.4 (22.5, 32.9)	33.2 (27.3, 39.7)	11.2 (8.8, 14.1)	28.3 (23.5, 34.5)
Sub-Sharan Africa	Sudan	53.3 (43.6. 62.8)	31.5 (23.9, 40.1)	5.8 (3.9, 8.5)	9.4 (7.5, 11.8)
Central Asia, Middle East, and north Africa	Syria	21.0 (17.7, 24.8)	54.1 (49.5, 58.6)	4.0 (3.2, 5.0)	20.9 (17.3, 25.0)
Sub-Sharan Africa	Tanzania	35.2 (30.5, 40.2)	27.3 (24.6, 30.2)	12.6 (10.9, 14.6)	24.9 (20.6, 29.8)
East and southeast Asia	Thailand	14.2 (11.5, 17.4)	75.4 (72.4, 79.0)	4.2 (3.0, 5.8)	6.3 (5.1, 7.7)
East and southeast Asia	Timor-Leste	15.9 (13.9, 18.2)	58.0 (55.2, 60.8)	6.4 (5.5, 7.3)	19.7 (17.0, 22.6)
Oceania	Tokelau	19.4 (13.5, 27.1)	34.2 (17.2, 56.5)	12.0 (8.2, 17.2)	34.5 (19.7, 52.9)
Oceania	Tonga	48.5 (44.8-52.2)	27.8 (24.2, 31.8)	7.4 (6.0, 9.0)	16.3 (14.6, 18.1)
Latin America and Caribbean	Trinidad and Tobago	30.1 (24.2, 36.6)	41.9 (36.6, 47.4)	8.1 (6.1, 10.6)	20.0 (17.2, 23.1)
Oceania	Tuvalu	44.0 (44.0, 44.0)	19.4 (19.4, 19.4)	9.1 (9.1, 9.1)	27.5 (27.5, 27.5)
Central Asia, Middle East and north Africa	United Arab Emirates	32.7 (27.4, 38.4)	39.8 (35.1, 44.7)	7.4 (5.9, 9.2)	20.1 (17.5, 23.0)
Latin America and Caribbean	Uruguay	12.6 (10.9, 14.4)	52.5 (49.5, 55.5)	8.3 (6.6, 10.3)	26.6 (24.8, 28.5)
Oceania	Vanuatu	31.3 (24.4, 43.2)	43.0 (34.1, 52.4)	6.8 (5.0, 9.3)	17.1 (12.7, 22.6)
East and southeast Asia	Vietnam	3.5 (2.3, 5.1)	92.0 (88.9, 93.8)	1.7 (1.3, 2.3)	2.8 (2.1, 3.8)

Oceania	Wallis and Futuna	9.6 (7.7, 12.0)	77.3 (73.6, 80.7)	7.2 (5.3, 9.8)	5.8 (4.3, 7.8)
Central Asia, Middle East and north Africa	Yemen	53.8 (46.4, 61.0)	20.1 (17.1, 23.5)	6.3 (5.1, 7.8)	19.8 (15.5, 25.1)
